# Supplementary material for: Electronic Interactions Between the Receptor-Binding Domain of Omicron Variants and Angiotensin-Converting Enzyme 2: A Novel Amino Acid–Amino Acid Bond Pair Concept
Source: Molecules. 2025 May 6;30(9):2061. doi: 10.3390/molecules30092061 (PMC12073306; doi:10.3390/molecules30092061)
Supplement: Supplementary file 1 [file molecules-30-02061-s001.zip › molecules-3529515-supplementary.pdf]

Supplementary Materials for Electronic Interactions Between the Receptor-Binding Domain of Omicron Variants and Angiotensin-Converting Enzyme 2: A Novel Amino Acid–Amino Acid Bond Pair Concept

Puja Adhikari[1]*, Bahaa Jawad[1,2] and Wai-Yim Ching[1]

1. Department of Physics and Astronomy, University of Missouri-Kansas City, Kansas City, MO 64110, USA.
2. Department of Applied Sciences, University of Technology, Baghdad 10066, Iraq

Corresponding Author: Puja Adhikari

Wai-Yim Ching:	chingw@umkc.edu	ORCID: 0000-0001-7738-8822
Puja Adhikari:	paz67@umkc.edu	ORCID: 0000-0002-5591-6505
Bahaa Jawad:	bahaa.a.jawad@uotechnology.edu.iq	ORCID: 0000-0001-6252-3999

Content

Table S1: Comparison of AABP units between WT and OV for all mutation sites. AABP is in units of electrons (e^-)

Figure S1: Comparison of shape changes in the AABPU for the site 440 across five models (a) WT, (b) OV BA.1, (c) OV BA.2, (d) OV BA.5, (e) OV XBB.1.16. In each model, the cyan color surface represents the site 440, the yellow surface indicates nearest neighbors (NN) residue, and the red surface denotes non-local (NL) residue. In the ball and stick representation, atoms are colored as follows: grey - carbon (C) , red – oxygen (O), white – hydrogen (H), blue – nitrogen (N).

Figure S2: Total AABP for the mutation sites for WT, OV BA.1, OV BA.2, OV BA.5, and OV XBB.1.16.

Figure S3: Total AABP for amino acids sequence from 333 to 526 from RBD for **(a)** WT, **(b)** BA.1, **(c)** BA.2, **(d)** BA.5, **(e)** XBB.1.16. The mutated amino acids are marked with black arrows.

Figure S4: Total AABP for amino acids sequence number from 19-88, 319-365 ACE2 for **(a)** WT, **(b)** BA.1, **(c)** BA.2, **(d)** BA.5, **(e)** XBB.1.16. The dashed lines show the break in sequence number.

Figure S5: Standard deviation in the AABP value for the (a) NN and (b) NL considering RBD of all five interface models—WT, OV BA.1, OV BA.2, OV BA.5, and OV XBB.1.16.

Figure S6: Standard deviation in the AABP value for the (a) NN and (b) NL considering ACE2 of all five interface models—WT, OV BA.1, OV BA.2, OV BA.5, and OV XBB.1.16.

Table S2: Partial Charge for all residues in RBD of Wild Type.

Table S3: Partial Charge for all residues in ACE2 of Wild Type.

Table S4: Partial Charge for all residues in RBD of Omicron Variant BA.1.

Table S5: Partial Charge for all residues in ACE2 of Omicron Variant BA.1.

Table S6: Partial Charge for all residues in RBD of Omicron Variant BA.2.

Table S7: Partial Charge for all residues in ACE2 of Omicron Variant BA.2.

Table S8: Partial Charge for all residues in RBD of Omicron Variant BA.5.

Table S9: Partial Charge for all residues in ACE2 of Omicron Variant BA.5.

Table S10: Partial Charge for all residues in RBD of Omicron Variant XBB.1.16.

Table S11: Partial Charge for all residues in ACE2 of Omicron Variant XBB.1.16.

Table S1: Comparison of AABP units between WT and OV for all mutation sites. AABP is in unit of electrons (e-).

Sites	AA Seq No	AABP (NN)	AABP (NL)	AABP (O···H)	AABP (N···H)	No Of NL AAs	WT/OV
339	G339	0.9894	0.0429	0.0604	0.0092	5	**WT**
	D339	1.0018	0.1092	0.1164	0.0092	5	**OV BA.1**
	D339	0.9990	0.1120	0.1204	0.0094	5	**OV BA.2**
	D339	1.0022	0.1189	0.1270	0.0097	5	**OV BA.5**
	H339	0.9847	0.0229	0.0369	0.0107	4	**OV XBB.1.16**
346	R346	0.9544	0.1043	0.1166	0.0082	5	**WT**
	R346	0.9326	0.0824	0.0992	0.0076	5	**OV BA.1**
	R346	0.9338	0.0813	0.0987	0.0079	5	**OV BA.2**
	R346	0.933	0.0795	0.0971	0.0077	5	**OV BA.5**
	T346	0.9306	0.0038	0.0245	0.0082	5	**OV XBB.1.16**
368	L368	0.9463	0.0126	0.0281	0.0107	10	**WT**
	L368	0.9521	0.0268	0.038	0.0077	9	**OV BA.1**
	L368	0.9584	0.0378	0.0512	0.0069	9	**OV BA.2**
	L368	0.9521	0.0345	0.0491	0.0078	8	**OV BA.5**
	I368	0.9384	0.0299	0.0438	0.0062	9	**OV XBB.1.16**
371	S371	0.9687	0.0586	0.0699	0.0072	5	**WT**
	L371	0.9037	0.0234	0.0415	0.0038	6	**OV BA.1**
	F371	0.9079	0.0220	0.0414	0.0035	4	**OV BA.2**
	F371	0.9065	0.0207	0.0398	0.0042	5	**OV BA.5**
	F371	0.9062	0.002	0.0197	0.0055	3	**OV XBB.1.16**
373	S373	0.9319	0.0768	0.0924	0.0080	3	**WT**
	P373	1.0075	0.0027	0.0180	0.0032	3	**OV BA.1**
	P373	1.0054	0.0026	0.0176	0.0033	3	**OV BA.2**
	P373	1.0037	0.0027	0.0174	0.0033	3	**OV BA.5**
	P373	1.0094	0.0017	0.0135	0.0028	3	**OV XBB.1.16**
375	S375	0.9978	0.0297	0.1065	0.0037	6	**WT**
	F375	1.0808	0.0545	0.1967	0.0029	6	**OV BA.1**
	F375	0.8862	0.0445	0.0632	0.0046	5	**OV BA.2**
	F375	0.8885	0.0384	0.0579	0.0046	5	**OV BA.5**

	F375	0.8961	0.0407	0.0548	0.0063	6	**OV XBB.1.16**
376	T376	1.0586	0.1896	0.2447	0.0059	7	**WT**
	T376	1.1623	0.1674	0.2892	0.005	5	**OV BA.1**
	A376	0.9570	0.0471	0.0611	0.0067	5	**OV BA.2**
	A376	0.9554	0.0446	0.0590	0.0066	5	**OV BA.5**
	A376	0.9603	0.0674	0.0821	0.0078	6	**OV XBB.1.16**
405	D405	0.9894	0.3132	0.3102	0.0109	5	**WT**
	D405	0.9466	0.1802	0.1874	0.0121	6	**OV BA.1**
	N405	0.9887	0.1294	0.1359	0.0120	5	**OV BA.2**
	N405	0.9895	0.1410	0.1471	0.0121	4	**OV BA.5**
	N405	0.9601	0.0608	0.0802	0.0092	4	**OV XBB.1.16**
408	R408	0.9291	0.1828	0.1914	0.0108	4	**WT**
	R408	0.9365	0.1987	0.2003	0.0109	5	**OV BA.1**
	S408	0.9313	0.0679	0.0852	0.0059	4	**OV BA.2**
	S408	0.9696	0.0768	0.0906	0.0087	5	**OV BA.5**
	S408	0.9746	0.0342	0.0531	0.0080	3	**OV XBB.1.16**
417	K417	1.0137	0.3739	0.1140	0.0092	9	**WT**
	N417	1.0282	0.0773	0.0867	0.0079	8	**OV BA.1**
	N417	1.0204	0.0708	0.0824	0.0078	7	**OV BA.2**
	N417	1.0243	0.0760	0.0864	0.0076	8	**OV BA.5**
	N417	1.0328	0.0692	0.0783	0.0076	7	**OV XBB.1.16**
440	N440	0.9191	0.0037	0.0306	0.0048	2	**WT**
	K440	0.9185	0.2796	0.0339	0.0057	3	**OV BA.1**
	K440	0.9229	0.2792	0.0332	0.0060	4	**OV BA.2**
	K440	0.9289	0.2788	0.0338	0.0065	4	**OV BA.5**
	K440	0.9317	0.0037	0.0278	0.0097	2	**OV XBB.1.16**
445	V445	1.0831	0.0006	0.1058	0.0046	3	**WT**
	V445	0.9852	0.0129	0.0352	0.0082	4	**OV BA.1**
	V445	0.9701	0.0102	0.0322	0.0083	4	**OV BA.2**
	V445	0.9558	0.0092	0.0322	0.0085	4	**OV BA.5**
	P445	1.0469	0.0043	0.0176	0.0082	4	**OV XBB.1.16**
446	G446	0.9719	0.0521	0.0705	0.0047	4	**WT**
	S446	0.9368	0.0582	0.0760	0.0067	3	**OV BA.1**
	G446	0.9344	0.0202	0.0389	0.01	2	**OV BA.2**
	G446	0.9976	0.1302	0.1476	0.0108	3	**OV BA.5**
	S446	0.9482	0.0353	0.0598	0.0080	3	**OV XBB.1.16**
452	L452	0.9825	0.0462	0.0595	0.0050	9	**WT**
	L452	0.9961	0.0512	0.0639	0.005	9	**OV BA.1**
	L452	0.9917	0.0512	0.0631	0.0052	9	**OV BA.2**
	R452	0.9879	0.0648	0.0762	0.0046	9	**OV BA.5**
	L452	0.984	0.0481	0.0594	0.0058	9	**OV XBB.1.16**
460	N460	1.0820	0.0630	0.1225	0.0055	4	**WT**

	N460	1.058	0.0766	0.135	0.0052	4	**OV BA.1**
	N460	1.066	0.0767	0.1343	0.0059	4	**OV BA.2**
	N460	1.0934	0.0756	0.1395	0.0057	4	**OV BA.5**
	K460	0.9786	0.2751	0.0251	0.0059	6	**OV XBB.1.16**
477	S477	0.9523	0.0129	0.0392	0.0048	2	**WT**
	N477	0.9383	0.2088	0.2123	0.0041	5	**OV BA.1**
	N477	0.9440	0.2065	0.2106	0.0042	4	**OV BA.2**
	N477	0.9445	0.2049	0.2088	0.0043	3	**OV BA.5**
	N477	0.9241	0.1744	0.1841	0.0035	3	**OV XBB.1.16**
478	T478	1.0476	0.0024	0.0179	0.0050	3	**WT**
	K478	1.0141	0.0636	0.0176	0.0041	4	**OV BA.1**
	K478	1.0163	0.0719	0.0217	0.0043	4	**OV BA.2**
	K478	1.0175	0.0201	0.0250	0.0047	5	**OV BA.5**
	R478	1.0219	0.0041	0.0165	0.0045	3	**OV XBB.1.16**
484	E484	0.9280	0.2419	0.2437	0.0042	4	**WT**
	A484	0.9255	0.0052	0.0296	0.0038	2	**OV BA.1**
	A484	0.9289	0.0037	0.0294	0.0037	2	**OV BA.2**
	A484	0.9287	0.0027	0.0285	0.0034	2	**OV BA.5**
	A484	0.9339	0.0039	0.0297	0.0039	2	**OV XBB.1.16**
486	F486	0.9345	0.0026	0.0177	0.0048	5	**WT**
	F486	0.9419	0.0609	0.0184	0.0047	6	**OV BA.1**
	F486	0.9396	0.0647	0.0188	0.0048	6	**OV BA.2**
	V486	0.9311	0.0081	0.0210	0.0050	4	**OV BA.5**
	P486	1.0147	0.0056	0.0107	0.0048	5	**OV XBB.1.16**
490	F490	1.0376	0.0886	0.0884	0.0044	7	**WT**
	F490	1.0819	0.0764	0.0773	0.004	6	**OV BA.1**
	F490	1.0833	0.075	0.0761	0.0037	6	**OV BA.2**
	F490	1.0178	0.0071	0.0138	0.0038	7	**OV BA.5**
	S490	1.0230	0.0875	0.0875	0.0057	5	**OV XBB.1.16**
493	Q493	0.9655	0.2478	0.2479	0.0079	8	**WT**
	R493	1.0706	0.2770	0.3185	0.0055	11	**OV BA.1**
	R493	1.0759	0.2764	0.3197	0.0054	11	**OV BA.2**
	Q493	0.9698	0.0683	0.0906	0.0057	7	**OV BA.5**
	Q493	1.0722	0.0812	0.1351	0.0101	9	**OV XBB.1.16**
496	G496	0.9764	0.0668	0.0820	0.0069	6	**WT**
	S496	0.9283	0.0841	0.0981	0.0052	7	**OV BA.1**
	G496	0.9378	0.0333	0.0517	0.0079	5	**OV BA.2**
	G496	0.9406	0.0389	0.0606	0.0079	6	**OV BA.5**
	G496	0.9961	0.0174	0.0335	0.0109	7	**OV XBB.1.16**
498	Q498	1.0828	0.1938	0.1791	0.0061	14	**WT**
	R498	1.0518	0.2391	0.2143	0.0079	14	**OV BA.1**
	R498	1.0589	0.2785	0.2539	0.0085	14	**OV BA.2**

	R498	1.0527	0.2890	0.2652	0.0086	14	**OV BA.5**
	R498	1.0634	0.2032	0.1793	0.0060	14	**OV XBB.1.16**
501	N501	0.9476	0.1860	0.1783	0.0042	9	**WT**
	Y501	0.9463	0.0827	0.0831	0.0049	9	**OV BA.1**
	Y501	0.9432	0.0744	0.0780	0.0050	10	**OV BA.2**
	Y501	0.9463	0.0728	0.0767	0.0046	10	**OV BA.5**
	Y501	0.9528	0.0826	0.0776	0.0059	10	**OV XBB.1.16**
505	Y505	1.0020	0.3388	0.1218	0.0066	10	**WT**
	H505	0.9754	0.1306	0.0924	0.0493	9	**OV BA.1**
	H505	0.9812	0.1599	0.1357	0.0294	9	**OV BA.2**
	H505	0.9852	0.1903	0.1317	0.0608	10	**OV BA.5**
	H505	0.9839	0.1315	0.1297	0.0083	10	**OV XBB.1.16**

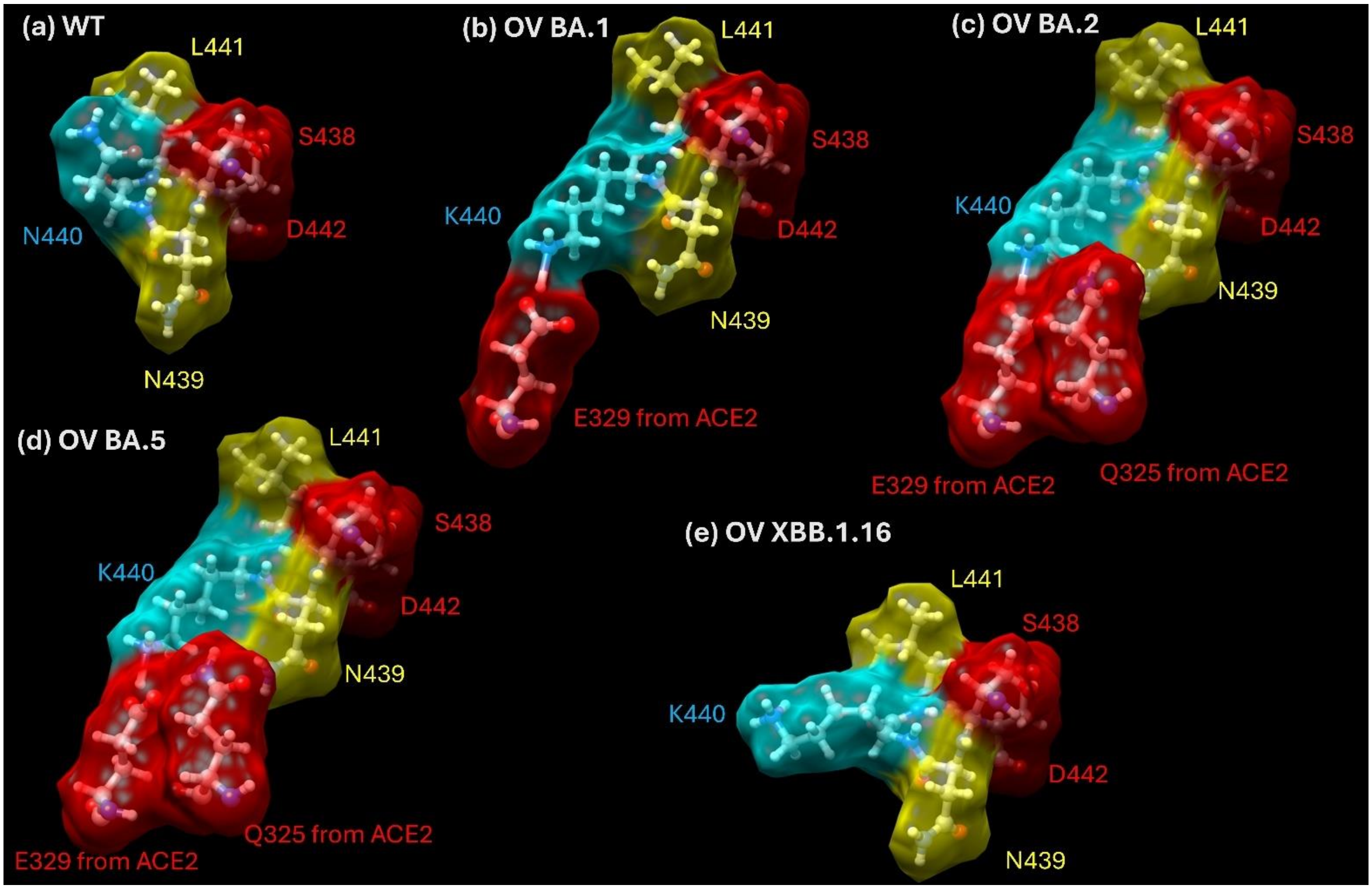

Figure S1: Comparison of shape changes in the AABPU for the site 440 across five models (a) WT, (b) OV BA.1, (c) OV BA.2, (d) OV BA.5, (e) OV XBB.1.16. In each model, the cyan color surface represents the site 440, the yellow surface indicates nearest neighbors (NN) residue, and the red surface denotes non-local (NL) residue. In the ball and stick representation, atoms are colored as follows: grey - carbon (C) , red – oxygen (O), white – hydrogen (H), blue – nitrogen (N).

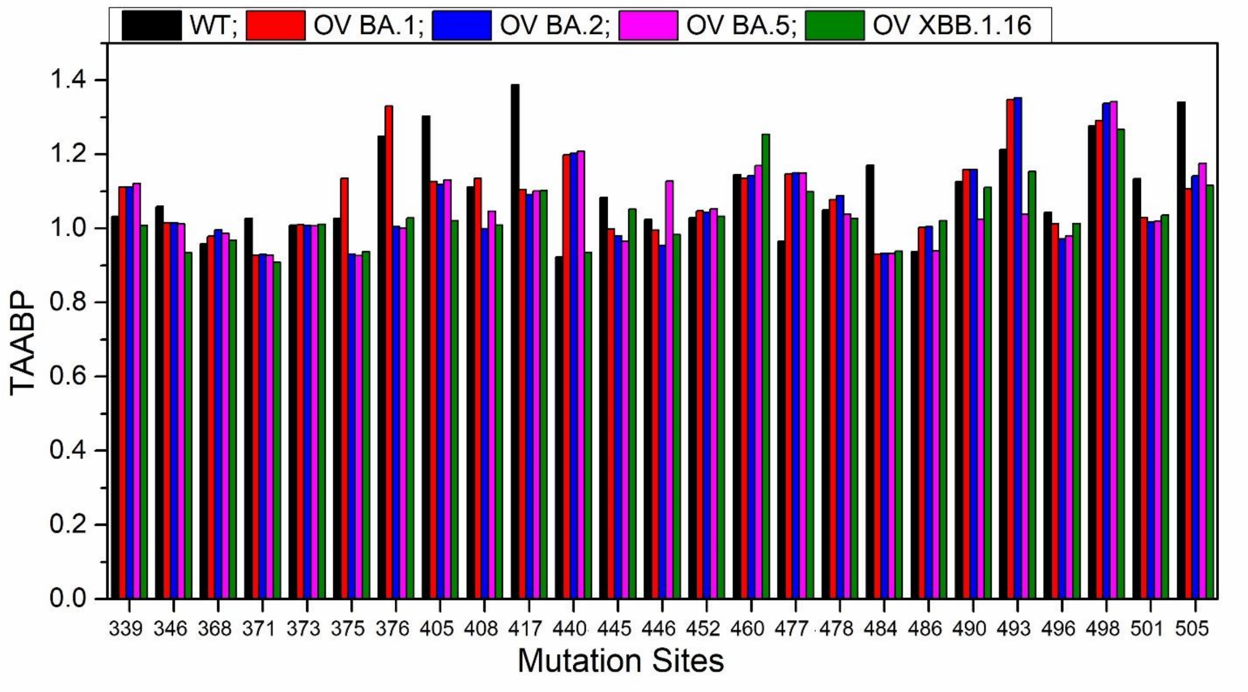

Figure S2: Total AABP for the mutation sites for WT, OV BA.1, OV BA.2, OV BA.5, and OV XBB.1.16.

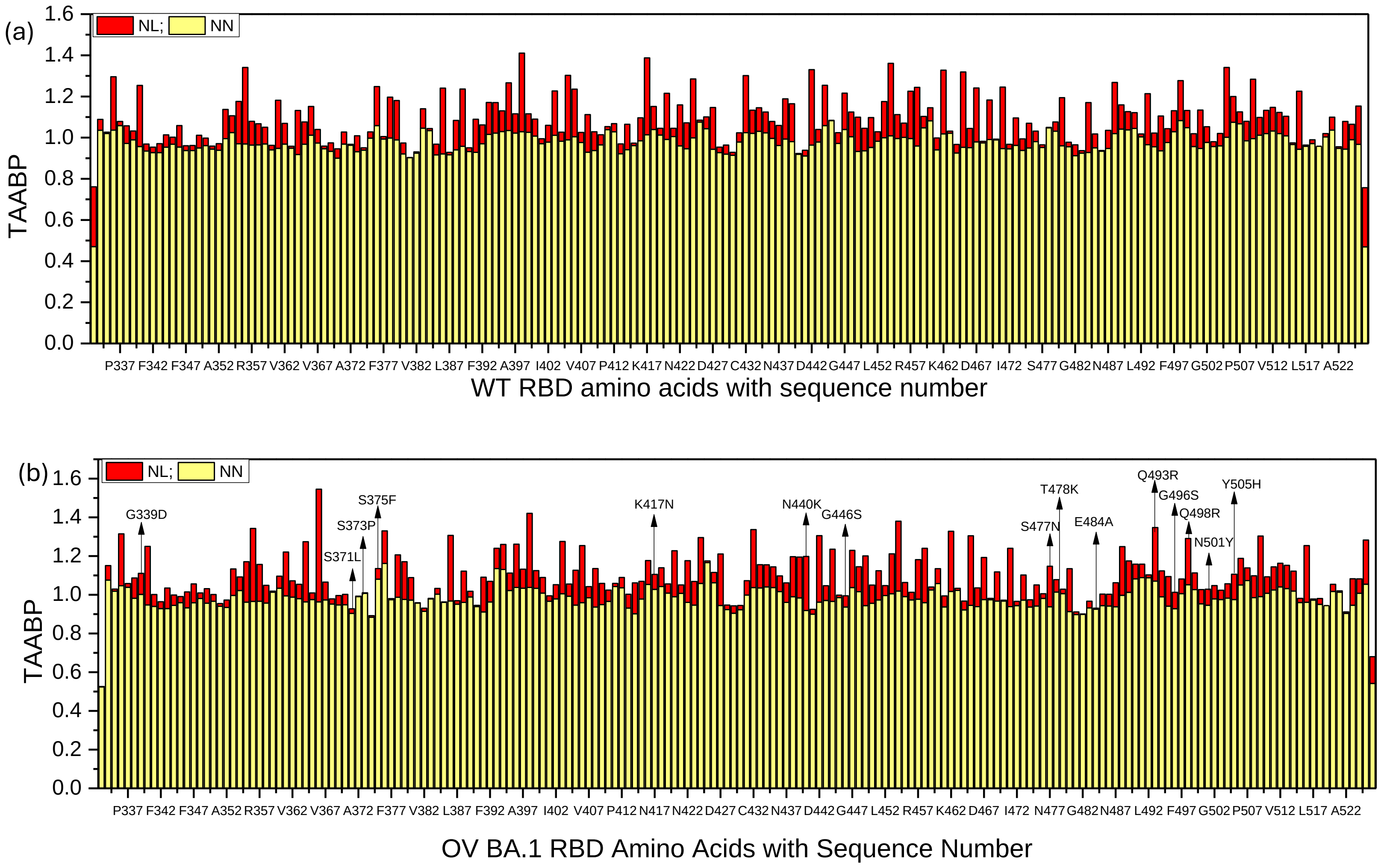
(a)
NL; NN
TAABP
1.6
1.4
1.2
1.0
0.8
0.6
0.4
0.2
0.0
P337 F342 F347 A352 R357 V362 V367 A372 F377 V382 L387 F392 A397 I402 V407 P412 K417 N422 D427 C432 N437 D442 G447 L452 R457 K462 D467 I472 S477 G482 N487 L492 F497 G502 P507 V512 L517 A522
WT RBD amino acids with sequence number
(b)
NL; NN
TAABP
G339D
S371L
S373P
S375F
K417N
N440K
G446S
S477N
T478K
E484A
Q493R
G496S
Q498R
N501Y
Y505H
P337 F342 F347 A352 R357 V362 V367 A372 F377 V382 L387 F392 A397 I402 V407 P412 N417 N422 D427 C432 N437 D442 G447 L452 R457 K462 D467 I472 N477 G482 N487 L492 F497 G502 P507 V512 L517 A522
OV BA.1 RBD Amino Acids with Sequence Number

(c)

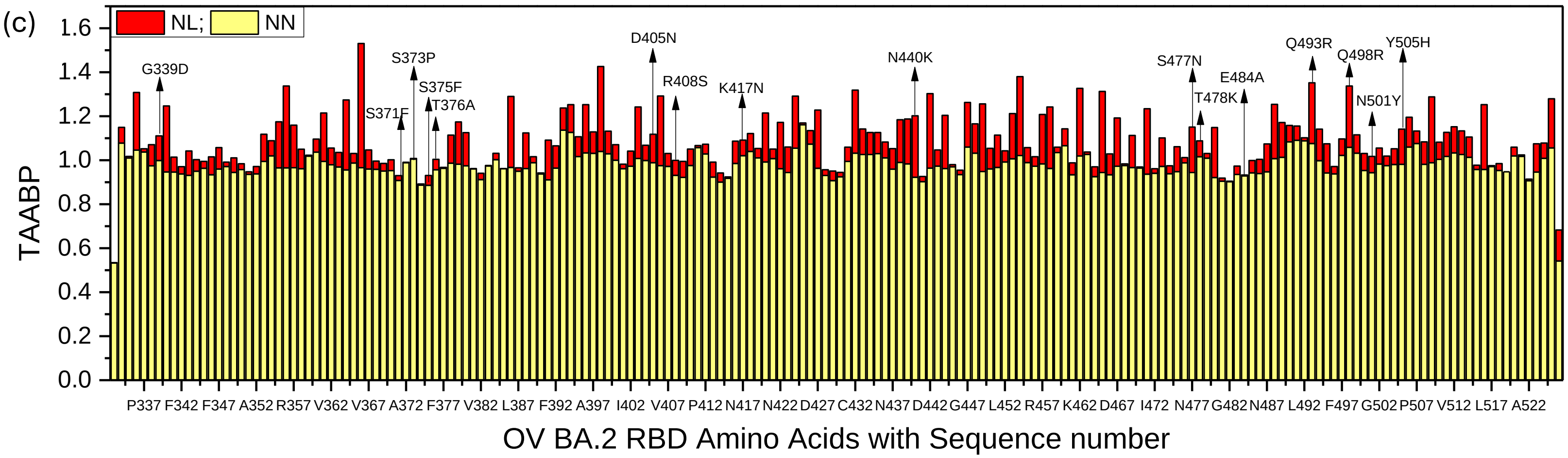
NL; NN
TAABP
G339D
S371F
S373P
S375F
T376A
D405N
R408S
K417N
N440K
S477N
T478K
E484A
Q493R
Q498R
N501Y
Y505H
P337 F342 F347 A352 R357 V362 V367 A372 F377 V382 L387 F392 A397 I402 V407 P412 N417 N422 D427 C432 N437 D442 G447 L452 R457 K462 D467 I472 N477 G482 N487 L492 F497 G502 P507 V512 L517 A522
OV BA.2 RBD Amino Acids with Sequence number

(d)

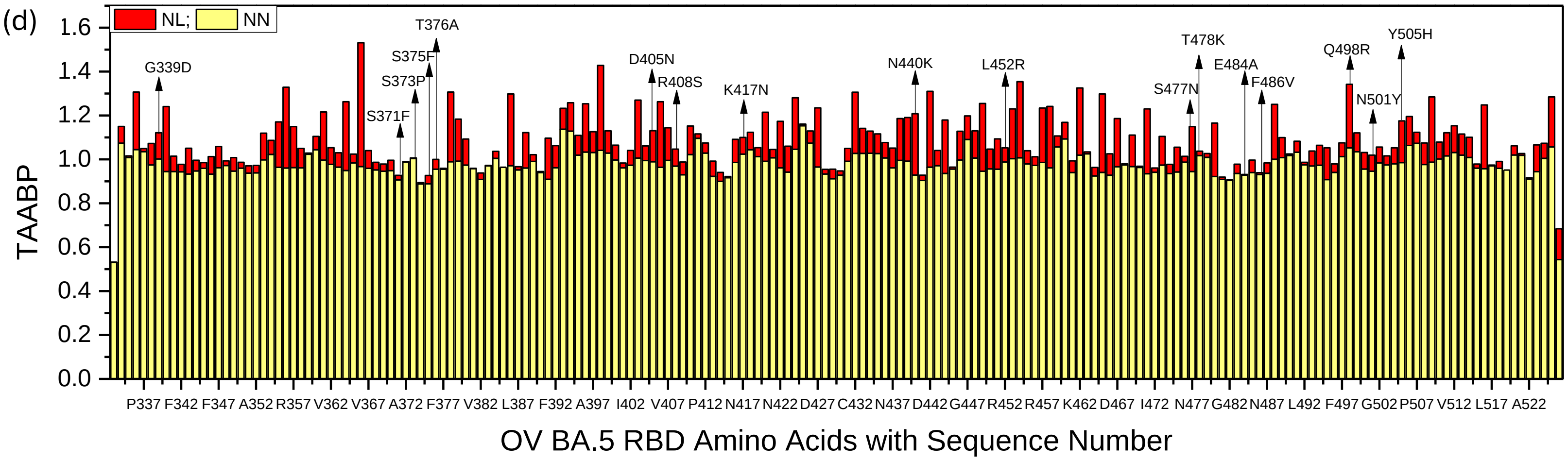
NL; NN
TAABP
G339D
S371F
S373P
S375F
T376A
D405N
R408S
K417N
N440K
L452R
S477N
T478K
E484A
F486V
Q498R
N501Y
Y505H
P337 F342 F347 A352 R357 V362 V367 A372 F377 V382 L387 F392 A397 I402 V407 P412 N417 N422 D427 C432 N437 D442 G447 R452 R457 K462 D467 I472 N477 G482 N487 L492 F497 G502 P507 V512 L517 A522
OV BA.5 RBD Amino Acids with Sequence Number

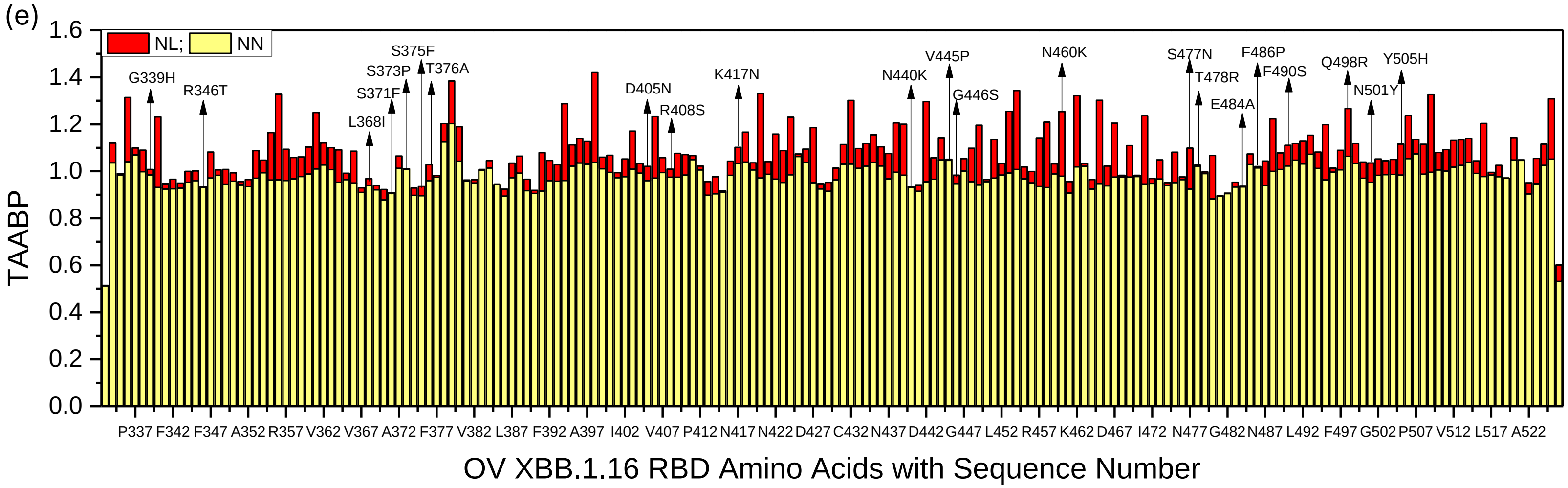

Figure S3: Total AABP for amino acids sequence from 333 to 526 from RBD for **(a)** WT, **(b)** BA.1, **(c)** BA.2, **(d)** BA.5, **(e)** XBB.1.16. The yellow and red color denotes contribution from nearest neighbor and non-local respectively. The mutated amino acids are marked with black arrows.

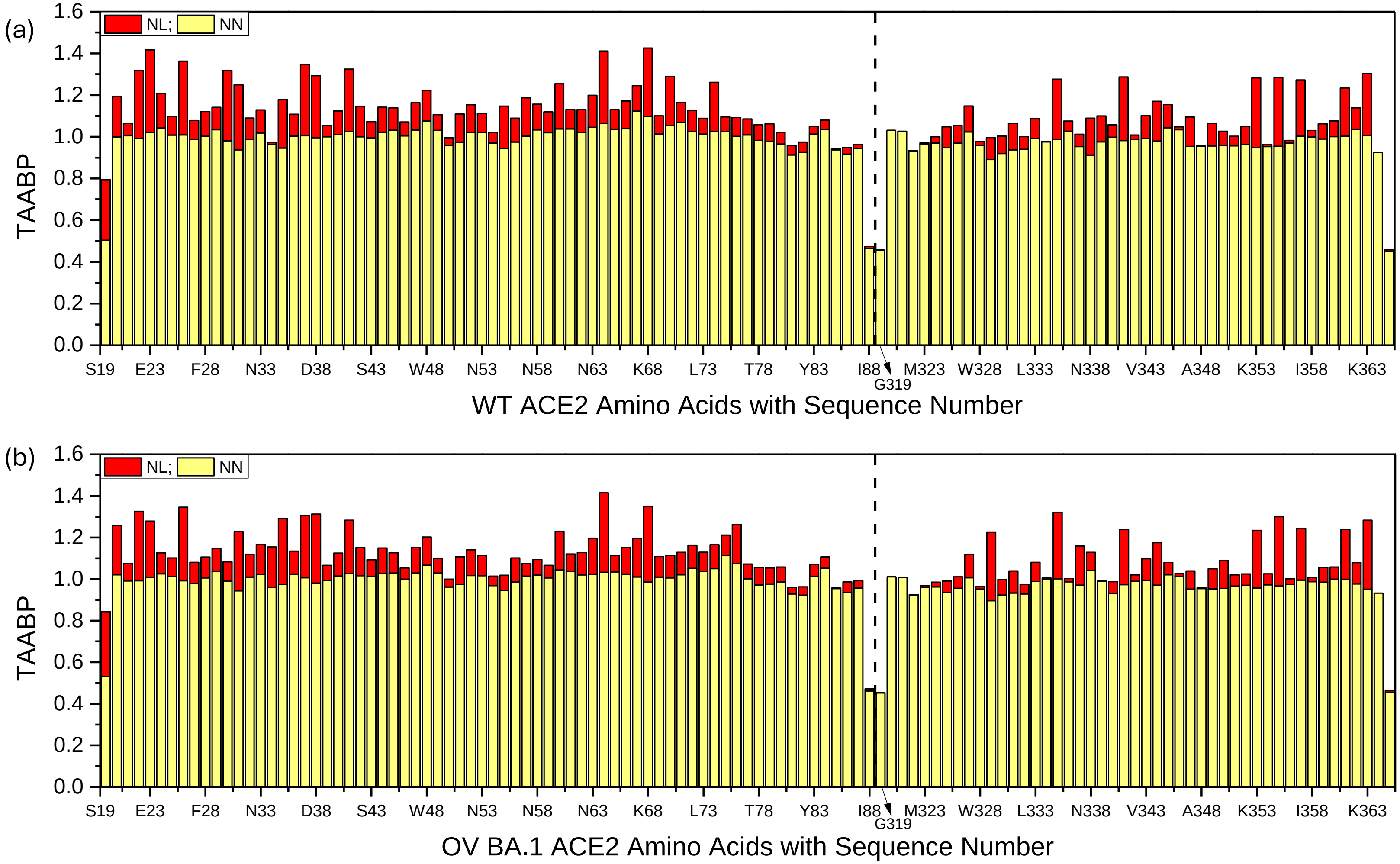
(a)
NL; NN
TAABP
S19 E23 F28 N33 D38 S43 W48 N53 N58 N63 K68 L73 T78 Y83 I88 G319 M323 W328 L333 N338 V343 A348 K353 I358 K363
WT ACE2 Amino Acids with Sequence Number
(b)
NL; NN
TAABP
S19 E23 F28 N33 D38 S43 W48 N53 N58 N63 K68 L73 T78 Y83 I88 G319 M323 W328 L333 N338 V343 A348 K353 I358 K363
OV BA.1 ACE2 Amino Acids with Sequence Number

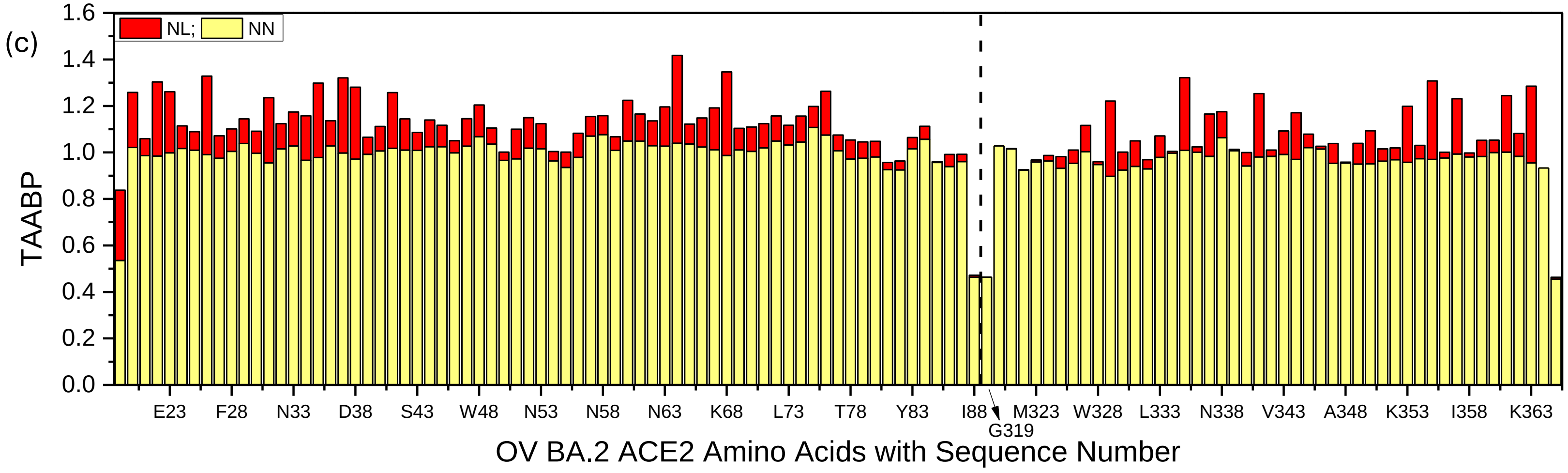

(c)
NL; NN
TAABP
1.6
1.4
1.2
1.0
0.8
0.6
0.4
0.2
0.0
E23 F28 N33 D38 S43 W48 N53 N58 N63 K68 L73 T78 Y83 I88 M323 W328 L333 N338 V343 A348 K353 I358 K363
G319
OV BA.2 ACE2 Amino Acids with Sequence Number

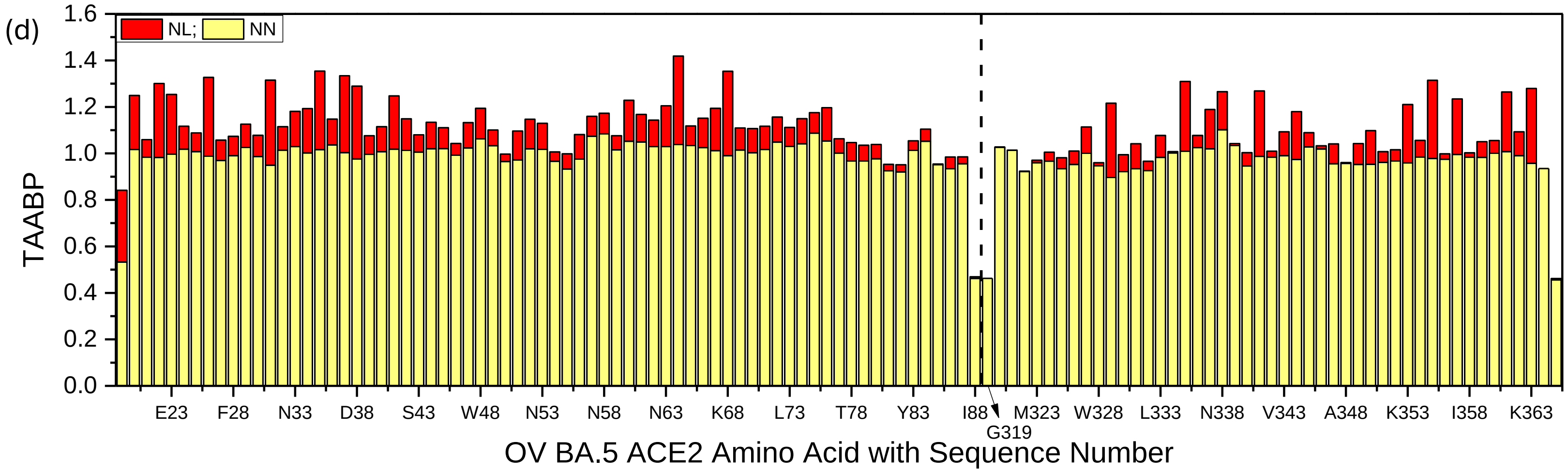

(d)
NL; NN
TAABP
1.6
1.4
1.2
1.0
0.8
0.6
0.4
0.2
0.0
E23 F28 N33 D38 S43 W48 N53 N58 N63 K68 L73 T78 Y83 I88 M323 W328 L333 N338 V343 A348 K353 I358 K363
G319
OV BA.5 ACE2 Amino Acid with Sequence Number

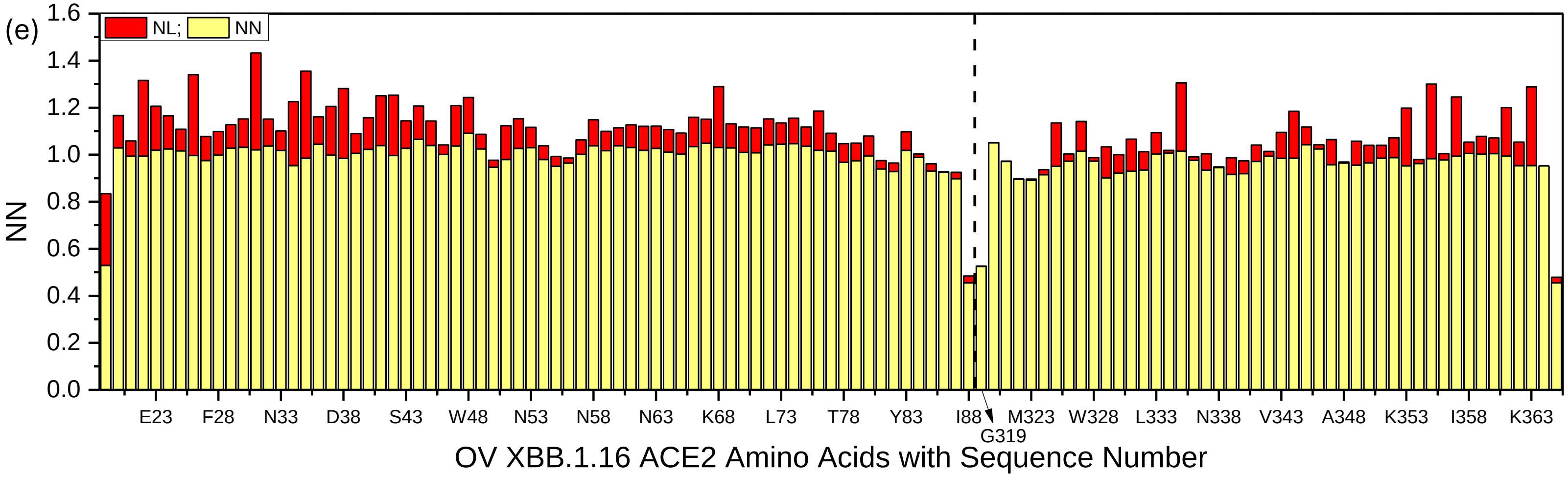

Figure S4: Total AABP for amino acids sequence number from 19-88, 319-365 ACE2 for **(a)** WT, **(b)** BA.1, **(c)** BA.2, **(d)** BA.5, **(e)** XBB.1.16. The yellow and red color denotes contribution from nearest neighbor and non-local respectively. The dashed lines show the break in sequence number.

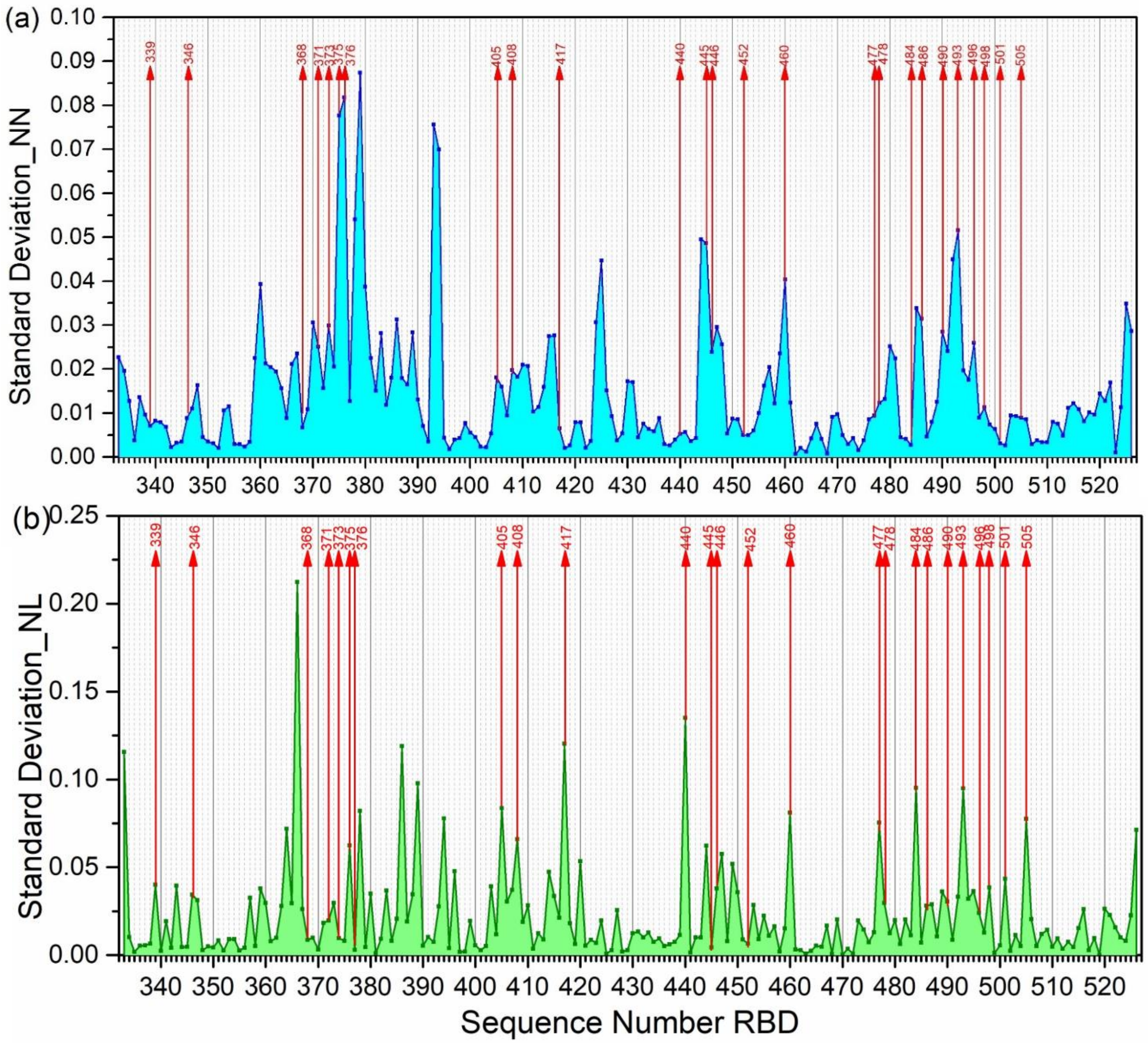

Figure S5: Standard deviation in the AABP value for the (a) NN and (b) NL considering RBD of all five interface models—WT, OV BA.1, OV BA.2, OV BA.5, and OV XBB.1.16.

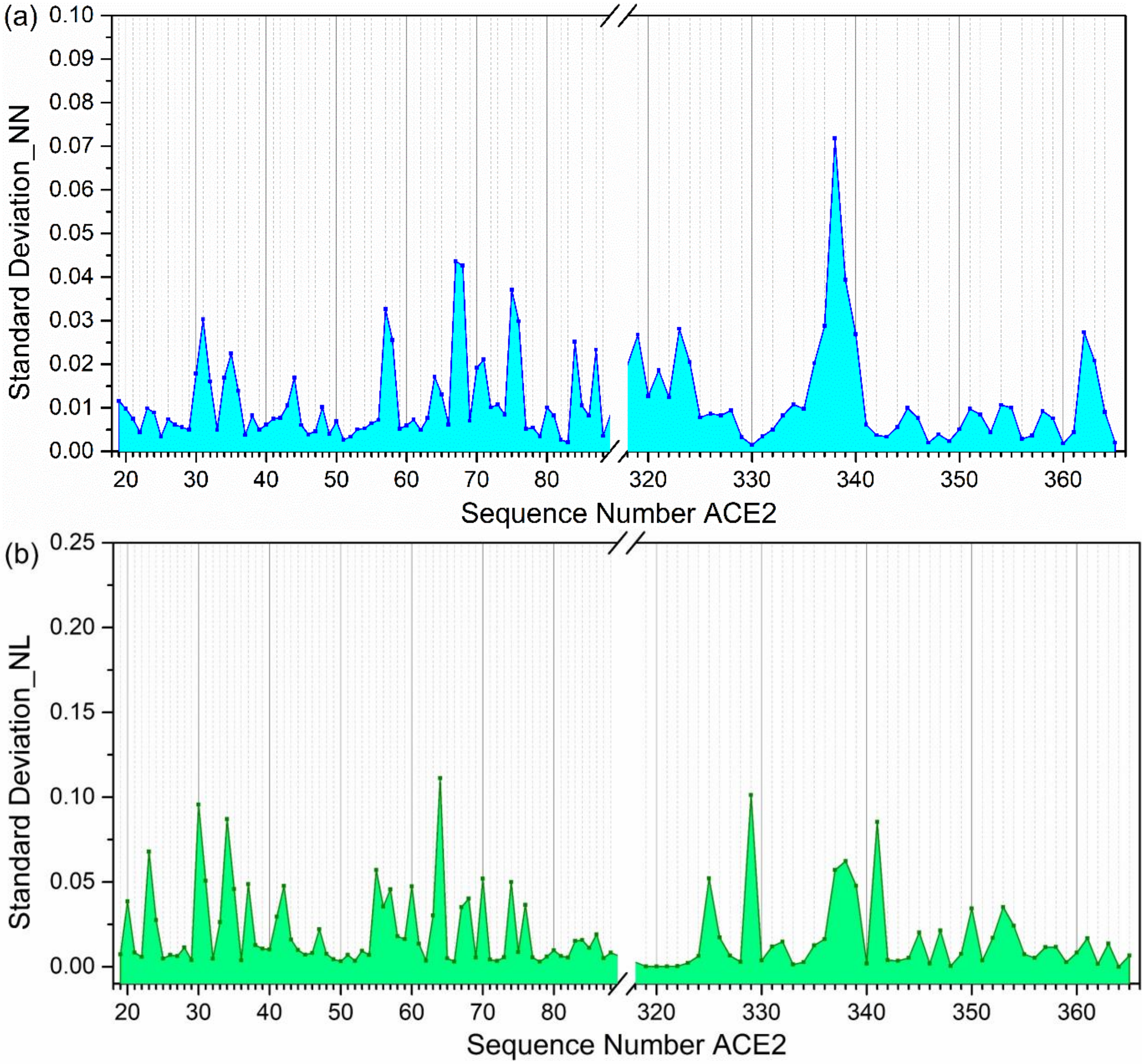

Figure S6: Standard deviation in the AABP value for the (a) NN and (b) NL considering ACE2 of all five interface models—WT, OV BA.1, OV BA.2, OV BA.5, and OV XBB.1.16.

Table S2: Partial Charge for all residues in RBD of Wild Type.

AA_Seq No	PC(e^-)	AA_Seq No	PC(e^-)	AA_Seq No	PC(e^-)	AA_Seq No	PC(e^-)
THR333	0.5095	VAL382	-0.0216	GLY431	-0.0397	CYS480	-0.0780
ASN334	-0.0895	SER383	-0.1057	CYS432	0.0794	ASN481	0.0742
LEU335	0.1586	PRO384	0.1712	VAL433	-0.0362	GLY482	-0.0650
CYS336	-0.1925	THR385	0.0503	ILE434	-0.0136	VAL483	-0.0204
PRO337	0.1947	LYS386	0.4481	ALA435	-0.0332	GLU484	-0.6220
PHE338	-0.0685	LEU387	-0.0379	TRP436	0.0165	GLY485	-0.0739
GLY339	0.1052	ASN388	-0.0098	ASN437	0.0787	PHE486	0.0479
GLU340	-0.4584	ASP389	-0.4820	SER438	-0.2249	ASN487	-0.0556
VAL341	-0.0630	LEU390	0.0010	ASN439	0.0722	CYS488	0.0640
PHE342	0.0006	CYS391	0.0075	ASN440	-0.0097	TYR489	0.0029
ASN343	-0.0503	PHE392	-0.0211	LEU441	-0.0079	PHE490	-0.0556
ALA344	0.0067	THR393	-0.0641	ASP442	-0.6751	PRO491	0.0190
THR345	0.0357	ASN394	0.0769	SER443	0.0268	LEU492	0.0657
ARG346	0.8657	VAL395	0.0169	LYS444	0.6133	GLN493	0.0179
PHE347	0.0062	TYR396	-0.1729	VAL445	0.1321	SER494	-0.0426
ALA348	0.0048	ALA397	0.0052	GLY446	0.0518	TYR495	-0.2134
SER349	0.0496	ASP398	-0.6474	GLY447	0.0302	GLY496	0.0651
VAL350	-0.0070	SER399	0.0628	ASN448	0.0367	PHE497	0.0290
TYR351	0.0640	PHE400	0.0163	TYR449	-0.1173	GLN498	0.0076
ALA352	-0.0507	VAL401	0.0243	ASN450	0.1604	PRO499	0.1777
TRP353	-0.0172	ILE402	-0.0482	TYR451	-0.1005	THR500	-0.0582
ASN354	-0.0056	ARG403	0.8156	LEU452	-0.0103	ASN501	-0.1179
ARG355	0.7201	GLY404	0.0411	TYR453	-0.1386	GLY502	0.0198
LYS356	0.4582	ASP405	-0.7020	ARG454	0.6865	VAL503	0.0432
ARG357	0.9138	GLU406	-0.7585	LEU455	-0.0542	GLY504	0.0978
ILE358	-0.1324	VAL407	-0.0345	PHE456	0.0523	TYR505	-0.4285
SER359	-0.0461	ARG408	0.8278	ARG457	0.7534	GLN506	-0.0327
ASN360	-0.0283	GLN409	-0.0129	LYS458	0.5508	PRO507	0.1401
CYS361	0.0475	ILE410	0.0027	SER459	-0.1130	TYR508	-0.0235
VAL362	-0.1121	ALA411	-0.1062	ASN460	0.0466	ARG509	0.6828
ALA363	0.0310	PRO412	0.1435	LEU461	0.0145	VAL510	0.0000
ASP364	-0.6505	GLY413	-0.0396	LYS462	0.3969	VAL511	-0.0757
TYR365	-0.0933	GLN414	-0.0141	PRO463	0.1279	VAL512	0.0124
SER366	-0.1012	THR415	0.0224	PHE464	-0.0516	LEU513	-0.0073
VAL367	-0.0266	GLY416	-0.0784	GLU465	-0.4326	SER514	0.0006
LEU368	-0.0320	LYS417	0.4925	ARG466	0.7840	PHE515	0.0926
TYR369	0.0032	ILE418	-0.0449	ASP467	-0.5976	GLU516	-0.6459
ASN370	-0.0533	ALA419	0.1032	ILE468	0.0373	LEU517	0.0041
SER371	-0.1003	ASP420	-0.8317	SER469	-0.1304	LEU518	-0.0311
ALA372	0.1457	TYR421	0.0312	THR470	0.0785	HIS519	0.0777
SER373	-0.0986	ASN422	-0.0338	GLU471	-0.5075	ALA520	-0.1090
PHE374	0.0410	TYR423	-0.1362	ILE472	-0.0048	PRO521	0.1204
SER375	0.0193	LYS424	0.6595	TYR473	-0.1001	ALA522	0.0756
THR376	0.0382	LEU425	-0.0128	GLN474	0.0242	THR523	-0.1123
PHE377	0.0469	PRO426	0.0835	ALA475	-0.0240	VAL524	-0.0442
LYS378	0.8193	ASP427	-0.6275	GLY476	0.0791	CYS525	0.0692
CYS379	0.0265	ASP428	-0.9855	SER477	0.0152	GLY526	-0.4830
TYR380	-0.0348	PHE429	-0.0156	THR478	-0.1250		
GLY381	0.0260	THR430	-0.0373	PRO479	0.1321		

Table S3: Partial Charge for all residues in ACE2 of Wild Type.

AA_Seq No	PC(e⁻)	AA_Seq No	PC(e⁻)	AA_Seq No	PC(e⁻)
SER19	0.6437	LYS68	0.5186	THR347	-0.1013
THR20	-0.1175	TRP69	0.0486	ALA348	0.0481
ILE21	0.0229	SER70	-0.0209	TRP349	1.8976
GLU22	-0.3930	ALA71	0.0761	ASP350	-0.8136
GLU23	-0.5492	PHE72	-0.0090	LEU351	-0.0889
GLN24	0.0609	LEU73	-0.0011	GLY352	-0.0317
ALA25	0.0015	LYS74	0.8158	LYS353	0.6069
LYS26	0.5116	GLU75	-0.9287	GLY354	0.0333
THR27	-0.0696	GLN76	-0.0058	ASP355	-0.5667
PHE28	0.0022	SER77	-0.0146	PHE356	-0.0336
LEU29	0.0397	THR78	-0.0296	ARG357	0.7186
ASP30	-0.4459	LEU79	-0.0599	ILE358	0.0274
LYS31	0.5729	ALA80	0.0017	LEU359	-0.0479
PHE32	-0.0808	GLN81	-0.0157	MET360	-0.0266
ASN33	-0.0155	MET82	-0.1079	CYS361	0.0823
HIS34	0.0782	TYR83	-0.0612	THR362	-0.0574
GLU35	-0.7229	PRO84	0.0566	LYS363	0.4979
ALA36	-0.0841	LEU85	0.1352	VAL364	0.0265
GLU37	-0.4862	GLN86	-0.1045	THR365	-1.0059
ASP38	-0.5892	GLU87	-1.0095		
LEU39	0.0272	ILE88	-0.9983		
PHE40	-0.0080	GLY319	1.0310		
TYR41	-0.0196	LEU320	-0.1838		
GLN42	0.0064	PRO321	0.1615		
SER43	-0.0064	ASN322	-0.0345		
SER44	-0.0359	MET323	0.0047		
LEU45	-0.0163	THR324	0.0455		
ALA46	0.0476	GLN325	-0.0815		
SER47	-0.0489	GLY326	0.0404		
TRP48	0.0249	PHE327	0.0515		
ASN49	0.0445	TRP328	2.0917		
TYR50	0.0300	GLU329	-0.8816		
ASN51	-0.0711	ASN330	-0.1086		
THR52	-0.0667	SER331	-0.0791		
ASN53	0.0356	MET332	0.0447		
ILE54	0.0171	LEU333	0.0206		
THR55	-0.1570	THR334	0.0935		
GLU56	-0.8149	ASP335	-0.6207		
GLU57	-0.7800	PRO336	0.0467		
ASN58	-0.0338	GLY337	-0.0266		
VAL59	0.0098	ASN338	0.1200		
GLN60	-0.0445	VAL339	0.0571		
ASN61	0.0529	GLN340	0.0166		
MET62	-0.0337	LYS341	0.6570		
ASN63	-0.0155	ALA342	0.0423		
ASN64	0.1983	VAL343	-0.0888		
ALA65	0.0966	CYS344	0.1231		
GLY66	-0.0378	HIS345	-0.1319		
ASP67	-0.7740	PRO346	0.1441		

Table S4: Partial Charge for all residues in RBD of Omicron Variant BA.1.

AA_Seq No	PC (e^-)	AA_Seq No	PC (e^-)	AA_Seq No	PC (e^-)	AA_Seq No	PC (e^-)
THR333	0.9139	VAL382	-0.0667	GLY431	-0.0462	CYS480	-0.1103
ASN334	-0.0385	SER383	-0.0188	CYS432	0.1055	ASN481	0.0561
LEU335	0.0932	PRO384	0.0397	VAL433	-0.0621	GLY482	-0.0046
CYS336	-0.1718	THR385	-0.0057	ILE434	-0.0093	VAL483	-0.0349
PRO337	0.1733	LYS386	0.5128	ALA435	0.0112	ALA484	0.0153
PHE338	-0.0767	LEU387	0.0842	TRP436	0.0571	GLY485	-0.0866
ASP339	-0.7636	ASN388	-0.1502	ASN437	0.0946	PHE486	0.1703
GLU340	-0.4423	ASP389	-0.9229	SER438	-0.2310	ASN487	0.0300
VAL341	-0.0532	LEU390	-0.0171	ASN439	0.0592	CYS488	0.0307
PHE342	-0.0107	CYS391	0.0005	LYS440	0.5675	TYR489	-0.0494
ASN343	-0.1300	PHE392	-0.0461	LEU441	-0.0247	PHE490	0.0257
ALA344	-0.0099	THR393	-0.0110	ASP442	-0.7335	PRO491	0.1590
THR345	0.0108	ASN394	0.1341	SER443	0.0321	LEU492	0.0072
ARG346	0.9492	VAL395	-0.0125	LYS444	0.6465	ARG493	0.7205
PHE347	0.0321	TYR396	-0.1649	VAL445	0.0919	SER494	-0.0727
ALA348	0.0816	ALA397	0.0189	SER446	0.0974	TYR495	-0.1534
SER349	0.0553	ASP398	-0.6458	GLY447	-0.0661	SER496	-0.1027
VAL350	0.0015	SER399	0.0512	ASN448	0.0469	PHE497	0.0298
TYR351	0.0719	PHE400	0.0098	TYR449	-0.1645	ARG498	0.6917
ALA352	-0.0671	VAL401	0.0352	ASN450	0.1534	PRO499	0.1123
TRP353	-0.0053	ILE402	-0.0454	TYR451	-0.1144	THR500	-0.0580
ASN354	-0.0008	ARG403	0.7277	LEU452	0.0015	TYR501	0.0325
ARG355	0.7134	GLY404	0.0054	TYR453	-0.1602	GLY502	0.0259
LYS356	0.4342	ASP405	-0.7574	ARG454	0.6797	VAL503	0.0363
ARG357	0.7949	GLU406	-0.7791	LEU455	-0.0220	GLY504	0.0938
ILE358	-0.0946	VAL407	-0.0090	PHE456	0.0490	HIS505	-0.0115
SER359	-0.0091	ARG408	0.7857	ARG457	0.7911	GLN506	-0.1013
ASN360	0.0273	GLN409	0.0018	LYS458	0.5457	PRO507	0.1408
CYS361	0.1378	ILE410	0.0369	SER459	-0.1310	TYR508	-0.0634
VAL362	-0.0802	ALA411	-0.0990	ASN460	0.0051	ARG509	0.6519
ALA363	-0.0726	PRO412	0.1309	LEU461	0.0090	VAL510	-0.0072
ASP364	-0.5263	GLY413	-0.0658	LYS462	0.4018	VAL511	-0.0549
TYR365	0.0592	GLN414	0.0711	PRO463	0.1374	VAL512	-0.0050
SER366	0.0636	THR415	-0.0142	PHE464	-0.0528	LEU513	0.0006
VAL367	-0.0134	GLY416	-0.0386	GLU465	-0.4633	SER514	-0.0223
LEU368	0.0360	ASN417	0.0586	ARG466	0.7988	PHE515	0.0940
TYR369	-0.0964	ILE418	-0.0182	ASP467	-0.6756	GLU516	-0.6917
ASN370	-0.0050	ALA419	0.0728	ILE468	0.0599	LEU517	0.0715
LEU371	-0.0428	ASP420	-0.8108	SER469	0.0044	LEU518	-0.0393
ALA372	-0.0830	TYR421	0.0357	THR470	0.0637	HIS519	0.0693
PRO373	0.0880	ASN422	-0.0431	GLU471	-0.5409	ALA520	-0.1435
PHE374	0.0906	TYR423	-0.1247	ILE472	-0.0206	PRO521	0.1380
PHE375	-0.0571	LYS424	0.6311	TYR473	-0.0981	ALA522	0.0253
THR376	-0.0158	LEU425	0.0131	GLN474	0.0916	THR523	-0.1571
PHE377	0.0661	PRO426	0.1379	ALA475	0.0236	VAL524	-0.0395
LYS378	0.7999	ASP427	-0.5583	GLY476	0.0257	CYS525	0.0660
CYS379	0.0498	ASP428	-0.9494	ASN477	0.1168	GLY526	-0.8132
TYR380	-0.1785	PHE429	-0.0391	LYS478	0.7745		
GLY381	0.1754	THR430	-0.0477	PRO479	0.1202		

Table S5: Partial Charge for all residues in ACE2 of Omicron Variant BA.1.

AA_Seq No	PC (e^-)	AA_Seq No	PC (e^-)	AA_Seq No	PC (e^-)
SER19	0.6716	LYS68	0.6061	THR347	-0.0331
THR20	-0.1309	TRP69	-0.0038	ALA348	0.0281
ILE21	-0.0042	SER70	-0.0269	TRP349	1.9117
GLU22	-0.3985	ALA71	-0.0263	ASP350	-0.7073
GLU23	-0.6702	PHE72	0.0223	LEU351	-0.0522
GLN24	0.0241	LEU73	0.0075	GLY352	-0.0746
ALA25	0.0020	LYS74	0.9900	LYS353	0.6637
LYS26	0.5053	GLU75	-0.8081	GLY354	-0.0485
THR27	-0.0709	GLN76	-0.0586	ASP355	-0.6037
PHE28	-0.0055	SER77	-0.0159	PHE356	-0.0317
LEU29	-0.0205	THR78	-0.0664	ARG357	0.7359
ASP30	-0.8206	LEU79	-0.0572	ILE358	0.0326
LYS31	0.6343	ALA80	-0.0186	LEU359	-0.0625
PHE32	-0.0742	GLN81	0.0283	MET360	-0.0011
ASN33	-0.0719	MET82	-0.0915	CYS361	0.0480
HIS34	0.1612	TYR83	-0.0654	THR362	-0.0785
GLU35	-0.6174	PRO84	0.0769	LYS363	0.4005
ALA36	-0.0502	LEU85	0.1614	VAL364	0.0133
GLU37	-0.5935	GLN86	-0.1645	THR365	-0.9916
ASP38	-0.5075	GLU87	-0.9530		
LEU39	-0.0215	ILE88	-1.0126		
PHE40	-0.0103	GLY319	1.0260		
TYR41	-0.0639	LEU320	-0.1455		
GLN42	0.0659	PRO321	0.1345		
SER43	0.0272	ASN322	-0.0327		
SER44	-0.0465	MET323	-0.0008		
LEU45	-0.0086	THR324	0.0591		
ALA46	0.0742	GLN325	-0.0415		
SER47	-0.0439	GLY326	0.0596		
TRP48	0.0277	PHE327	0.0237		
ASN49	0.0272	TRP328	0.1005		
TYR50	0.0332	GLU329	-0.5312		
ASN51	-0.0565	ASN330	-0.0751		
THR52	-0.0629	SER331	-0.0718		
ASN53	-0.0028	MET332	0.0332		
ILE54	0.0044	LEU333	0.0022		
THR55	-0.0658	THR334	0.0566		
GLU56	-0.8280	ASP335	-0.4705		
GLU57	-0.9176	PRO336	0.0504		
ASN58	0.0804	GLY337	0.0057		
VAL59	0.0061	ASN338	0.1262		
GLN60	-0.1295	VAL339	0.0490		
ASN61	0.0464	GLN340	-0.0701		
MET62	-0.0390	LYS341	0.7177		
ASN63	-0.0333	ALA342	0.0410		
ASN64	0.2391	VAL343	-0.0836		
ALA65	0.0976	CYS344	0.1358		
GLY66	-0.0310	HIS345	-0.1268		
ASP67	-0.7957	PRO346	0.0939		

Table S6: Partial Charge for all residues in RBD of Omicron Variant BA.2.

AA_Seq No	PC (e^-)	AA_Seq No	PC (e^-)	AA_Seq No	PC (e^-)	AA_Seq No	PC (e^-)
THR333	0.9001	VAL382	-0.0708	GLY431	-0.0301	CYS480	-0.1290
ASN334	-0.0174	SER383	-0.0281	CYS432	0.1142	ASN481	0.0697
LEU335	0.0900	PRO384	0.0355	VAL433	-0.0534	GLY482	-0.0085
CYS336	-0.1580	THR385	-0.0074	ILE434	-0.0168	VAL483	-0.0378
PRO337	0.1801	LYS386	0.5410	ALA435	0.0039	ALA484	0.0129
PHE338	-0.0936	LEU387	0.0833	TRP436	0.0549	GLY485	-0.0874
ASP339	-0.7626	ASN388	-0.1500	ASN437	0.0920	PHE486	0.1573
GLU340	-0.4303	ASP389	-0.9264	SER438	-0.2105	ASN487	0.0409
VAL341	-0.0332	LEU390	-0.0117	ASN439	0.0359	CYS488	0.0558
PHE342	0.0007	CYS391	-0.0149	LYS440	0.5648	TYR489	-0.0476
ASN343	-0.1370	PHE392	-0.0488	LEU441	-0.0188	PHE490	0.0080
ALA344	-0.0156	THR393	-0.0144	ASP442	-0.7400	PRO491	0.1774
THR345	0.0139	ASN394	0.1369	SER443	0.0365	LEU492	0.0085
ARG346	0.9460	VAL395	-0.0203	LYS444	0.6644	ARG493	0.7180
PHE347	0.0328	TYR396	-0.1717	VAL445	0.0685	SER494	-0.0873
ALA348	0.0886	ALA397	0.0222	GLY446	0.0350	TYR495	-0.1036
SER349	0.0263	ASP398	-0.6349	GLY447	-0.0294	GLY496	-0.0109
VAL350	0.0059	SER399	0.0400	ASN448	0.0879	PHE497	0.0592
TYR351	0.0530	PHE400	0.0103	TYR449	-0.2147	ARG498	0.6403
ALA352	-0.0602	VAL401	0.0371	ASN450	0.1477	PRO499	0.1234
TRP353	0.0147	ILE402	-0.0341	TYR451	-0.1090	THR500	-0.0626
ASN354	-0.0046	ARG403	0.7599	LEU452	0.0015	TYR501	0.0174
ARG355	0.7029	GLY404	0.0009	TYR453	-0.1646	GLY502	0.0411
LYS356	0.4269	ASN405	0.0635	ARG454	0.6902	VAL503	0.0402
ARG357	0.7716	GLU406	-0.6496	LEU455	-0.0166	GLY504	0.1135
ILE358	-0.0892	VAL407	-0.0084	PHE456	0.0457	HIS505	-0.1190
SER359	-0.0123	SER408	-0.0401	ARG457	0.7616	GLN506	-0.1035
ASN360	0.0322	GLN409	-0.0282	LYS458	0.5516	PRO507	0.1500
CYS361	0.1287	ILE410	0.0698	SER459	-0.1402	TYR508	-0.0609
VAL362	-0.0717	ALA411	-0.0786	ASN460	0.0103	ARG509	0.6680
ALA363	-0.0768	PRO412	0.1172	LEU461	0.0203	VAL510	-0.0070
ASP364	-0.4731	GLY413	-0.0623	LYS462	0.3875	VAL511	-0.0400
TYR365	0.0360	GLN414	-0.0522	PRO463	0.1327	VAL512	-0.0232
SER366	0.0210	THR415	-0.0203	PHE464	-0.0602	LEU513	0.0029
VAL367	-0.0405	GLY416	-0.0962	GLU465	-0.4310	SER514	-0.0207
LEU368	0.0260	ASN417	0.0448	ARG466	0.8033	PHE515	0.0930
TYR369	-0.0682	ILE418	-0.0122	ASP467	-0.6695	GLU516	-0.6762
ASN370	-0.0152	ALA419	0.0785	ILE468	0.0505	LEU517	0.0687
PHE371	-0.0175	ASP420	-0.8203	SER469	0.0069	LEU518	-0.0368
ALA372	-0.1032	TYR421	0.0341	THR470	0.0562	HIS519	0.0679
PRO373	0.0943	ASN422	-0.0481	GLU471	-0.5426	ALA520	-0.1437
PHE374	0.0682	TYR423	-0.1241	ILE472	-0.0243	PRO521	0.1369
PHE375	-0.1271	LYS424	0.6401	TYR473	-0.0978	ALA522	0.0262
ALA376	-0.0514	LEU425	0.0164	GLN474	0.0987	THR523	-0.1485
PHE377	0.0591	PRO426	0.1105	ALA475	-0.0054	VAL524	-0.0413
LYS378	0.8965	ASP427	-0.5497	GLY476	0.0328	CYS525	0.0719
CYS379	0.0490	ASP428	-0.9322	ASN477	0.1135	GLY526	-0.8061
TYR380	-0.1974	PHE429	-0.0508	LYS478	0.7723		
GLY381	0.1654	THR430	-0.0482	PRO479	0.1186		

Table S7: Partial Charge for all residues in ACE2 of Omicron Variant BA.2.

AA_Seq No	PC (e^-)	AA_Seq No	PC (e^-)	AA_Seq No	PC (e^-)
SER19	0.6828	LYS68	0.6019	THR347	-0.0272
THR20	-0.1317	TRP69	0.0054	ALA348	0.027
ILE21	-0.0055	SER70	-0.0274	TRP349	1.9151
GLU22	-0.4092	ALA71	-0.0293	ASP350	-0.7047
GLU23	-0.6642	PHE72	0.0221	LEU351	-0.0409
GLN24	0.0079	LEU73	0.0063	GLY352	-0.0777
ALA25	0.0064	LYS74	0.9893	LYS353	0.7041
LYS26	0.5187	GLU75	-0.8096	GLY354	-0.061
THR27	-0.0691	GLN76	-0.0561	ASP355	-0.6192
PHE28	-0.0052	SER77	-0.0069	PHE356	-0.0266
LEU29	-0.0196	THR78	-0.0624	ARG357	0.7498
ASP30	-0.8179	LEU79	-0.0526	ILE358	0.0379
LYS31	0.6483	ALA80	-0.022	LEU359	-0.0711
PHE32	-0.0707	GLN81	0.0231	MET360	0.0031
ASN33	-0.0848	MET82	-0.0894	CYS361	0.059
HIS34	0.1543	TYR83	-0.0649	THR362	-0.0715
GLU35	-0.6359	PRO84	0.0867	LYS363	0.4156
ALA36	-0.0374	LEU85	0.1632	VAL364	0.0183
GLU37	-0.5384	GLN86	-0.1708	THR365	-0.9949
ASP38	-0.5319	GLU87	-0.9573		
LEU39	-0.0311	ILE88	-1.0127		
PHE40	-0.0048	GLY319	1.0175		
TYR41	-0.0548	LEU320	-0.1466		
GLN42	0.0784	PRO321	0.1427		
SER43	0.0222	ASN322	-0.0223		
SER44	-0.0393	MET323	-0.0056		
LEU45	-0.0058	THR324	0.0582		
ALA46	0.0752	GLN325	-0.0459		
SER47	-0.0441	GLY326	0.053		
TRP48	0.0229	PHE327	0.0395		
ASN49	0.0305	TRP328	0.0933		
TYR50	0.0399	GLU329	-0.5156		
ASN51	-0.0444	ASN330	-0.0819		
THR52	-0.0757	SER331	-0.0866		
ASN53	-0.0164	MET332	0.0571		
ILE54	0.009	LEU333	-0.0014		
THR55	-0.06	THR334	0.0343		
GLU56	-0.8629	ASP335	-0.489		
GLU57	-0.7951	PRO336	0.0476		
ASN58	0.0229	GLY337	0.0073		
VAL59	-0.0028	ASN338	0.1553		
GLN60	-0.1219	VAL339	0.0474		
ASN61	0.0269	GLN340	-0.0816		
MET62	-0.034	LYS341	0.6998		
ASN63	-0.0303	ALA342	0.0365		
ASN64	0.2388	VAL343	-0.0864		
ALA65	0.0909	CYS344	0.1342		
GLY66	-0.0339	HIS345	-0.1364		
ASP67	-0.8052	PRO346	0.0976		

Table S8: Partial Charge for all residues in RBD of Omicron Variant BA.5.

AA_Seq No	PC (e^-)	AA_Seq No	PC (e^-)	AA_Seq No	PC (e^-)	AA_Seq No	PC (e^-)
THR333	0.9055	VAL382	-0.0711	GLY431	-0.0202	CYS480	-0.1441
ASN334	-0.0271	SER383	-0.0344	CYS432	0.1147	ASN481	0.0743
LEU335	0.0919	PRO384	0.0374	VAL433	-0.0527	GLY482	-0.0122
CYS336	-0.1441	THR385	-0.0058	ILE434	-0.0144	VAL483	-0.0377
PRO337	0.1821	LYS386	0.5390	ALA435	0.0051	ALA484	0.0156
PHE338	-0.0933	LEU387	0.0900	TRP436	0.0477	GLY485	-0.0778
ASP339	-0.7552	ASN388	-0.1467	ASN437	0.0889	VAL486	0.0591
GLU340	-0.4246	ASP389	-0.9312	SER438	-0.2029	ASN487	-0.0415
VAL341	-0.0377	LEU390	-0.0079	ASN439	0.0301	CYS488	0.0555
PHE342	-0.0024	CYS391	-0.0168	LYS440	0.5606	TYR489	0.0544
ASN343	-0.1413	PHE392	-0.0429	LEU441	-0.0189	PHE490	0.0317
ALA344	-0.0108	THR393	-0.0174	ASP442	-0.7333	PRO491	0.0337
THR345	0.0069	ASN394	0.1337	SER443	0.0485	LEU492	0.0421
ARG346	0.9486	VAL395	-0.0218	LYS444	0.6591	GLN493	-0.0281
PHE347	0.0327	TYR396	-0.1781	VAL445	0.0236	SER494	-0.0754
ALA348	0.0936	ALA397	0.0281	GLY446	0.0770	TYR495	-0.1633
SER349	0.0207	ASP398	-0.6341	GLY447	0.0566	GLY496	-0.0038
VAL350	0.0046	SER399	0.0338	ASN448	0.0353	PHE497	0.0686
TYR351	0.0915	PHE400	0.0103	TYR449	-0.2276	ARG498	0.5946
ALA352	-0.0572	VAL401	0.0336	ASN450	0.1441	PRO499	0.1287
TRP353	0.0111	ILE402	-0.0292	TYR451	-0.1129	THR500	-0.0644
ASN354	0.0073	ARG403	0.7133	ARG452	0.9555	TYR501	0.0183
ARG355	0.7054	GLY404	-0.0064	TYR453	-0.2094	GLY502	0.0403
LYS356	0.4318	ASN405	0.0587	ARG454	0.6779	VAL503	0.0372
ARG357	0.7741	GLU406	-0.6359	LEU455	-0.0009	GLY504	0.1065
ILE358	-0.0893	VAL407	-0.0059	PHE456	0.0363	HIS505	-0.0584
SER359	-0.0242	SER408	0.0294	ARG457	0.7310	GLN506	-0.0986
ASN360	0.0436	GLN409	-0.0046	LYS458	0.5536	PRO507	0.1617
CYS361	0.1290	ILE410	0.0807	SER459	-0.1398	TYR508	-0.0668
VAL362	-0.0798	ALA411	-0.0273	ASN460	0.0382	ARG509	0.6664
ALA363	-0.0795	PRO412	0.1172	LEU461	0.0286	VAL510	-0.0092
ASP364	-0.4753	GLY413	-0.0613	LYS462	0.3813	VAL511	-0.0388
TYR365	0.0310	GLN414	-0.0517	PRO463	0.1386	VAL512	-0.0278
SER366	0.0171	THR415	-0.0140	PHE464	-0.0563	LEU513	0.0070
VAL367	-0.0354	GLY416	-0.1081	GLU465	-0.4368	SER514	-0.0295
LEU368	0.0242	ASN417	0.0436	ARG466	0.8080	PHE515	0.0962
TYR369	-0.0733	ILE418	-0.0013	ASP467	-0.6717	GLU516	-0.6737
ASN370	-0.0082	ALA419	0.0875	ILE468	0.0475	LEU517	0.0609
PHE371	-0.0213	ASP420	-0.8156	SER469	0.0021	LEU518	-0.0357
ALA372	-0.0931	TYR421	0.0373	THR470	0.0482	HIS519	0.0731
PRO373	0.0830	ASN422	-0.0531	GLU471	-0.5298	ALA520	-0.1441
PHE374	0.0610	TYR423	-0.1291	ILE472	-0.0315	PRO521	0.1345
PHE375	-0.1157	LYS424	0.6323	TYR473	-0.0959	ALA522	0.0284
ALA376	-0.0411	LEU425	0.0030	GLN474	0.1026	THR523	-0.1438
PHE377	0.0629	PRO426	0.1181	ALA475	0.0252	VAL524	-0.0486
LYS378	0.6473	ASP427	-0.5365	GLY476	0.0269	CYS525	0.0681
CYS379	0.0552	ASP428	-0.9385	ASN477	0.1233	GLY526	-0.8068
TYR380	-0.1407	PHE429	-0.0547	LYS478	0.8496		
GLY381	0.1614	THR430	-0.0458	PRO479	0.1306		

Table S9: Partial Charge for all residues in ACE2 of Omicron Variant BA.5.

AA_Seq No	PC (e^-)	AA_Seq No	PC (e^-)	AA_Seq No
SER19	0.6739	LYS68	0.5981	THR347
THR20	-0.1339	TRP69	0.0072	ALA348
ILE21	0.0023	SER70	-0.0280	TRP349
GLU22	-0.4122	ALA71	-0.0303	ASP350
GLU23	-0.6701	PHE72	0.0094	LEU351
GLN24	0.0083	LEU73	0.0084	GLY352
ALA25	0.0054	LYS74	0.9867	LYS353
LYS26	0.5117	GLU75	-0.8436	GLY354
THR27	-0.0674	GLN76	-0.0564	ASP355
PHE28	-0.0227	SER77	-0.0067	PHE356
LEU29	-0.0270	THR78	-0.0679	ARG357
ASP30	-0.8334	LEU79	-0.0519	ILE358
LYS31	0.4160	ALA80	-0.0245	LEU359
PHE32	-0.0572	GLN81	0.0282	MET360
ASN33	-0.0802	MET82	-0.0774	CYS361
HIS34	0.2229	TYR83	-0.0768	THR362
GLU35	-0.4825	PRO84	0.0819	LYS363
ALA36	-0.0246	LEU85	0.1591	VAL364
GLU37	-0.5362	GLN86	-0.1645	THR365
ASP38	-0.5371	GLU87	-0.9576	
LEU39	-0.0313	ILE88	-1.0152	
PHE40	-0.0084	GLY319	1.0171	
TYR41	-0.0425	LEU320	-0.1452	
GLN42	0.0832	PRO321	0.1457	
SER43	0.0201	ASN322	-0.0268	
SER44	-0.0421	MET323	-0.0041	
LEU45	-0.0120	THR324	0.0398	
ALA46	0.0655	GLN325	-0.0411	
SER47	-0.0403	GLY326	0.0590	
TRP48	0.0228	PHE327	0.0385	
ASN49	0.0264	TRP328	0.0864	
TYR50	0.0505	GLU329	-0.4992	
ASN51	-0.0471	ASN330	-0.0903	
THR52	-0.0737	SER331	-0.0899	
ASN53	-0.0162	MET332	0.0584	
ILE54	0.0075	LEU333	-0.0011	
THR55	-0.0563	THR334	0.0387	
GLU56	-0.8635	ASP335	-0.5183	
GLU57	-0.7943	PRO336	0.0527	
ASN58	0.0312	GLY337	-0.0086	
VAL59	0.0060	ASN338	0.1881	
GLN60	-0.1213	VAL339	0.0280	
ASN61	0.0234	GLN340	-0.0725	
MET62	-0.0473	LYS341	0.6782	
ASN63	-0.0364	ALA342	0.0416	
ASN64	0.2491	VAL343	-0.0864	
ALA65	0.0894	CYS344	0.1237	
GLY66	-0.0331	HIS345	-0.1345	
ASP67	-0.8031	PRO346	0.0924	

Table S10: Partial Charge for all residues in RBD of Omicron Variant XBB.1.16.

AA_Seq No	PC (e⁻)	AA_Seq	PC (e⁻)	AA_Seq	PC (e⁻)	AA_Seq	PC (e⁻)
THR333	0.9141	VAL382	-0.0417	GLY431	0.0077	CYS480	-0.0744
ASN334	-0.0129	SER383	-0.0037	CYS432	0.0800	ASN481	0.0316
LEU335	0.1213	PRO384	0.0367	VAL433	-0.0216	GLY482	-0.0130
CYS336	-0.2186	THR385	0.0479	ILE434	-0.0293	VAL483	-0.0212
PRO337	0.1700	LYS386	0.9698	ALA435	-0.0259	ALA484	0.0124
PHE338	-0.0413	LEU387	-0.0719	TRP436	0.0429	GLY485	-0.1438
HIS339	0.1028	ASN388	0.0235	ASN437	0.0833	PRO486	0.1257
GLU340	-0.5370	ASP389	-1.0037	SER438	-0.2507	ASN487	-0.0026
VAL341	-0.0406	LEU390	-0.0755	ASN439	0.0751	CYS488	0.0415
PHE342	-0.0375	CYS391	0.0085	LYS440	0.9844	TYR489	0.0785
ASN343	-0.0310	PHE392	-0.0931	LEU441	0.0160	SER490	-0.0944
ALA344	0.0075	THR393	-0.0123	ASP442	-0.7058	PRO491	0.0794
THR345	0.0440	ASN394	-0.0434	SER443	-0.0049	LEU492	0.0773
THR346	0.0406	VAL395	-0.0483	LYS444	0.7357	GLN493	0.0931
PHE347	0.0457	TYR396	0.0236	PRO445	0.2594	SER494	-0.0674
ALA348	0.0829	ALA397	0.0174	SER446	-0.0315	TYR495	-0.1491
SER349	0.0637	ASP398	-0.5946	GLY447	-0.0323	GLY496	0.1087
VAL350	-0.0103	SER399	0.0571	ASN448	-0.0534	PHE497	0.0227
TYR351	0.0640	PHE400	-0.0056	TYR449	-0.1846	ARG498	0.7368
ALA352	-0.0599	VAL401	0.0324	ASN450	0.0542	PRO499	0.1254
TRP353	0.0110	ILE402	-0.0387	TYR451	-0.1244	THR500	-0.0297
ASN354	0.0211	ARG403	0.8737	LEU452	-0.0175	TYR501	0.0629
ARG355	0.7245	GLY404	0.0018	TYR453	-0.2692	GLY502	0.0053
LYS356	0.4981	ASN405	0.0104	ARG454	0.7169	VAL503	0.0719
ARG357	0.8259	GLU406	-0.7758	LEU455	-0.0127	GLY504	0.1107
ILE358	-0.0876	VAL407	-0.0409	PHE456	-0.0249	HIS505	-0.1401
SER359	0.0405	SER408	0.1150	ARG457	0.7004	GLN506	-0.1262
ASN360	0.0597	GLN409	0.0164	LYS458	0.5535	PRO507	0.1488
CYS361	0.1034	ILE410	0.0296	SER459	-0.0264	TYR508	-0.0805
VAL362	-0.0548	ALA411	-0.0722	LYS460	0.5875	ARG509	0.6279
ALA363	-0.0672	PRO412	0.0882	LEU461	-0.0095	VAL510	-0.0102
ASP364	-0.7688	GLY413	-0.0706	LYS462	0.3806	VAL511	-0.0363
TYR365	-0.0059	GLN414	-0.0235	PRO463	0.1208	VAL512	-0.0388
SER366	-0.1329	THR415	-0.0097	PHE464	-0.0564	LEU513	0.0198
VAL367	-0.0086	GLY416	-0.0535	GLU465	-0.4571	SER514	-0.0471
ILE368	-0.0780	ASN417	0.0986	ARG466	0.8345	PHE515	0.0938
TYR369	0.0461	ILE418	-0.0690	ASP467	-0.6600	GLU516	-0.7980
ASN370	0.0225	ALA419	0.1073	ILE468	0.0569	LEU517	0.0832
PHE371	-0.0119	ASP420	-0.5275	SER469	-0.0283	LEU518	-0.0482
ALA372	-0.1340	TYR421	-0.0066	THR470	0.0664	HIS519	0.0887
PRO373	0.1118	ASN422	-0.0654	GLU471	-0.5820	ALA520	-0.1613
PHE374	0.0036	TYR423	-0.1447	ILE472	-0.0041	PRO521	0.1728
PHE375	-0.0723	LYS424	0.7192	TYR473	-0.0383	ALA522	-0.0179
ALA376	-0.0561	LEU425	-0.0392	GLN474	0.0667	THR523	-0.0992
PHE377	0.0493	PRO426	0.0803	ALA475	0.0094	VAL524	-0.0442
LYS378	0.7790	ASP427	-0.5843	GLY476	0.0411	CYS525	0.1029
CYS379	0.0912	ASP428	-0.9474	ASN477	0.1020	GLY526	-0.8057
TYR380	-0.0384	PHE429	-0.0537	ARG478	0.8681		
GLY381	0.1080	THR430	-0.0316	PRO479	0.1537		

Table S11: Partial Charge for all residues in ACE2 of Omicron Variant XBB.1.16.

**AA_Seq No**	**PC (e^-)**	**AA_Seq No**	**PC (e^-)**	**AA_Seq No**	**PC (e^-)**
SER19	0.6954	LYS68	0.7852	THR347	-0.0585
THR20	-0.0384	TRP69	0.0388	ALA348	0.0410
ILE21	0.0270	SER70	-0.0344	TRP349	-0.0960
GLU22	-0.4333	ALA71	-0.0364	ASP350	-0.8093
GLU23	-0.7707	PHE72	-0.0273	LEU351	-0.0658
GLN24	0.0254	LEU73	0.0186	GLY352	-0.0438
ALA25	0.0072	LYS74	1.0080	LYS353	0.7319
LYS26	0.5190	GLU75	-0.9533	GLY354	-0.0037
THR27	-0.0753	GLN76	0.0503	ASP355	-0.5562
PHE28	-0.0038	SER77	0.0075	PHE356	0.0012
LEU29	-0.0377	THR78	-0.0643	ARG357	0.7229
ASP30	-0.8500	LEU79	-0.0600	ILE358	0.0147
LYS31	0.4269	ALA80	-0.0444	LEU359	-0.0428
PHE32	-0.0180	GLN81	0.0429	MET360	-0.0215
ASN33	0.0427	MET82	-0.0834	CYS361	0.0473
HIS34	0.2028	TYR83	-0.1579	THR362	-0.0978
GLU35	-0.5722	PRO84	0.1249	LYS363	0.3858
ALA36	-0.0380	LEU85	-0.0394	VAL364	0.0660
GLU37	-0.6991	GLN86	0.0755	THR365	0.9615
ASP38	-0.5885	GLU87	-0.9835		
LEU39	-0.0005	ILE88	0.9048		
PHE40	-0.0400	GLY319	-0.9020		
TYR41	-0.0144	LEU320	-0.1400		
GLN42	0.1762	PRO321	0.0664		
SER43	-0.0172	ASN322	0.0130		
SER44	-0.0191	MET323	-0.0034		
LEU45	-0.0116	THR324	0.0087		
ALA46	0.0774	GLN325	-0.1061		
SER47	-0.1145	GLY326	0.0651		
TRP48	0.0522	PHE327	0.0403		
ASN49	0.0593	TRP328	0.1092		
TYR50	0.0184	GLU329	-0.8558		
ASN51	-0.0870	ASN330	-0.1249		
THR52	-0.0863	SER331	-0.0809		
ASN53	0.0631	MET332	0.0027		
ILE54	0.0047	LEU333	0.0374		
THR55	-0.0106	THR334	0.0488		
GLU56	-0.9344	ASP335	-0.4828		
GLU57	-1.0081	PRO336	0.0607		
ASN58	0.0204	GLY337	-0.0001		
VAL59	0.0299	ASN338	0.0787		
GLN60	0.0201	VAL339	-0.0985		
ASN61	0.0149	GLN340	-0.0636		
MET62	-0.0258	LYS341	0.9548		
ASN63	0.0299	ALA342	0.0879		
ASN64	-0.0130	VAL343	-0.1214		
ALA65	0.0234	CYS344	0.1377		
GLY66	-0.0139	HIS345	-0.1129		
ASP67	-0.9535	PRO346	0.0952		
